# Professionals’ Recommended Strategies to Improve Australian Adolescents’ Knowledge of Nutrition and Food Systems

**DOI:** 10.3390/nu9080844

**Published:** 2017-08-07

**Authors:** Sanaz Sadegholvad, Heather Yeatman, Anne-Maree Parrish, Anthony Worsley

**Affiliations:** 1 School of Health and Society, Faculty of Social Sciences, University of Wollongong, Wollongong, NSW 2522, Australia; hyeatman@uow.edu.au (H.Y.); aparrish@uow.edu.au (A.-M.P.); 2 Centre for Physical Activity Research, School of Exercise and Nutrition Sciences, Deakin University, Burwood, VIC 3125, Australia; anthony.worsley@deakin.edu.au

**Keywords:** education strategies, nutrition, food systems, knowledge, Australian adolescents

## Abstract

Background: Education and policy measures within schools are valuable strategies to promote health. This study explored views of experienced food-related educators, researchers and policy-makers regarding their recommended strategies to improve Australian adolescents’ knowledge of nutrition and food systems (N&FS). Methods: Semi-structured interviews were conducted with twenty-one experienced food-related experts from across Australia. Interviews were conducted either by telephone or face-to-face. Recorded interviews were transcribed verbatim and analyzed thematically. Results: Five central themes and five sub-themes were identified from food professionals’ suggestions for best strategies to improve adolescents’ knowledge of N&FS. The central themes included: (1) specific improvements in schools’ core curricula; (2) pre-service and in-service training of school teachers about N&FS; (3) training students to develop a critical mind about N&FS issues; (4) multidisciplinary collaborations to improve school-based N&FS education; and (5) a supportive N&FS education environment for students. Conclusion and implication: These findings provide a guide for curriculum developers, educational policy developers, and food educators to incorporate the suggested N&FS strategies into Australian education programs in order to improve Australian adolescents’ knowledge and skills of N&FS issues. The results of this investigation also may assist the development of international N&FS curricula guides.

## 1. Introduction

Education and policy measures within school settings are valuable approaches to improve health. Schools offer the most effective and efficient environment to reach a large proportion of the community including young people, families, school staff and community members [1]. The positive effects of school-based food and nutrition education programs to improve students’ knowledge [2,3,4,5,6,7], attitudes [5,8], preferences [5,9], behavior [3,4,7,8] and self-efficiency [5,10] are reported in literature from around the world. The ultimate purpose of school-based nutrition education initiatives is to support reductions in the burdens of nutrition-related health problems. Unfortunately, the prevalence of obesity among children and adolescents has become a critical global health challenge [7] and poor dietary behaviors are the leading contributor to burden of disease. In high-income countries changes in dietary intakes have resulted in over-consumption of energy while diet quality remains poor [11] and in middle and low-income countries there has been a dietary transition toward increased consumption of refined carbohydrates, added sugars and fats [12].

Research indicates that healthy eating is associated with an increase in quality of life, life expectancy and decreases in nutrition-related chronic diseases such as cardiovascular disease, cancer and diabetes [13]. Eating habits and preferences are shaped from childhood and gradually become part of an individual’s routine [14]. One important strategy to assist youth to appreciate and develop healthy dietary behaviors is to offer nutrition education programs and supportive food environment in schools [13].

In addition to traditional nutrition education for students, which is primarily designed to foster healthy dietary behaviors, there also is a need to improve students’ knowledge of food systems and their capacities to think critically about the wider impacts of their food choices [15]. Food systems are defined as “the process of food production, distribution, preparation, and preservation; food use and consumption; the recycling and disposal of food wastes; and the systems that support this process including marketing, transportation, storage, and government services” [15]. Food production and consumption connects people and the environment [16]. It is important to promote among the public appropriate food choices that are supportive of sustaining natural resources, the environment and food systems, and knowledge of food systems has been recommended [15].

For decades experts have recommended food-related educational programs be incorporated into school curricula and delivered to enhance children’s understandings [17]. School-based education programs should teach students not only the required, broadly-based nutrition and food systems (N&FS) knowledge, but also the necessary skills such as making appropriate food choices, food preparation and food storage [17,18,19]. Nutrition education investigators have raised the need to use a wide range of teaching methods for school-based nutrition education programs [18,19]. Selected methods need to be based on learning objectives “from classroom discussions, work-sheets and keeping food records; to shopping exercises, tasting, creating or drama” [18]. In addition, the World Health Organization’s Information Series on School Health recommended a combination of various teaching methods for students to improve their knowledge, beliefs and skills [20].

Specific learning approaches to improve students’ nutrition knowledge or dietary behaviors are separately explored and recommended in the literature, such as integrating nutrition topics with other school subjects [6], food preparation and cooking activities [21,22], school gardens [9,23], and game-based innovations [24,25]. Carraway-Stage and colleagues also suggest that in order to increase students’ exposure to nutrition-related information in schools it is necessary to integrate nutrition topics within other subjects (such as science) [6].

Overall very few studies have focused on the identification of a full range of effective strategies to improve students’ knowledge of nutrition and food systems during formal schooling [19,26]. One study, a systematic review and meta-analysis of teaching approaches to promote healthy eating among elementary school students [26], identified cross-curricular strategies and experiential learning methods as the most effective ways to improve children’s dietary behaviors [26].

In previous work we have reported on an investigation of experts’ views of best strategies to improve Iranian adolescents’ knowledge of nutrition and food systems [19]. That study identified the need for a multi-levelled, systematic approach that included three key components of: policy; the process of education; and supportive environments. A wide range of strategies was suggested to improve adolescents knowledge of N&FS. Some of the identified strategies were: training school teachers, increasing skill-based and skill developing education programs at schools, developing critical thinking among students regarding food-related issues, and modifying inaccurate beliefs and traditions among Iranian students [19].

To the best of our knowledge, there is a paucity of research into efficient and effective methods to improve Australian students’ knowledge of N&FS during formal schooling. Therefore, this study aimed to develop a broad guide for strategies to improve Australian adolescents knowledge of nutrition and food systems.

## 2. Materials and Methods 

The study interviewed highly experienced food and education professionals who were engaged in various nutrition and food systems (N&FS) fields, including: home economists, nutritionists, dietitians, public health nutritionists, food scientists, environmental scientists and veterinary physicians from four States (New South Wales, Victoria, Queensland and Western Australia) and one Territory (Australian Capital Territory) of Australia. Purposive sampling method [27] was employed to select an appropriate group of prominent professionals from these fields. The inclusion criteria were: experienced academics from top ranked universities of Australia with long time contributions to food-related education programs and relevant research; acknowledged policymakers who were involved in community nutrition education programs; and experts with key roles in professional governmental and non-governmental organizations. To select appropriate academics, the first and second authors performed a thorough web-based investigation of staff profiles from all relevant schools or departments from major universities in Australia. Potential participants were selected who were experienced in young Australians’ food education programs or those who were involved in Australian food and nutrition education research. A similar web-based search was performed to identify a group of appropriate participants from Australian organizations who were active in nutrition education, consumer welfare, animal welfare and environmental sustainability. Further searches were performed in ‘Research gate’ and ‘LinkedIn’. The investigators developed a list of forty eligible experts. The objective was to stop recruitment once data saturation had occurred [28]. Ethics approval was granted from the Human Research Ethics Committee (Health and Medical) at the University of Wollongong (Approval No: HE12/277).

Sanaz Sadegholvad (SS) and Heather Yeatman (HY) invited potential participants via email for face-to-face or telephone-based interviews. The experts who agreed to participate were issued with the participant information sheet, consent form and the interview questions. Interview dates/times and location were arranged via email. Before commencing each interview, participants provided an informed written and/or audio recorded verbal consent.

SS conducted semi-structured interviews with each of the participants who participated in this study. Semi-structured interviewing methods [29] provided the opportunity for interviewees to express and expand their views on the best strategies to improve adolescents’ knowledge of N&FS. Interview questions were developed by the authors and were confirmed with a panel of four experienced academics with food education and research backgrounds the University of Wollongong. Three key open-ended questions were used for all interviews:

1. What are the best strategies to increase adolescents’ knowledge of nutrition and food systems?
2. What do you think could be done differently to increase their knowledge?
3. Do you have other suggestions for school-based N&FS education programs?

All the interviews were audio-recorded. The interviews were transcribed verbatim by a professional audio transcriber and reviewed and checked by the first author. Inductive thematic analysis [30,31] was conducted using manual coding and the Nvivo 10 software program. In an inductive thematic analysis the researchers do not try to code the data to fit a pre-established coding frame or the investigators’ analytic perceptions. This method of analysis is data driven [30].

Steps described by Baum and Clerk [30], and Vasmoradi and colleagues [31] guided the thematic analysis. Initially, the first author read and reviewed all the transcripts several times to become familiar with the collected data. Initial codes were generated from the features of the entire data set. Potential themes were generated from the coded data by the first author and were reviewed, re-reviewed and named by the first and second investigators. The reviews of transcripts continued until no new themes emerged from the entire data set. All authors reached consensus in relation to the labeled themes for the final report. Finally, a brief report of identified themes and details of each theme and sample quotes were developed to present the findings in a clear strategic manner [30,31]. 

## 3. Results

Data collection ended after interviewing twenty-one experts, when data saturation had occurred. All interviewees were experienced educators and/or prominent researchers from universities and/or governmental and non-governmental organizations. In addition some of the interviewees were involved in policymaking. Interviewees included three public health nutritionists, one public health expert, four dietitians, four nutritionists, four home economists, two food scientists, two veterinary physicians (experts in animal-sourced food production and animal welfare issues) and one environmental scientist.

**Figure 1:**
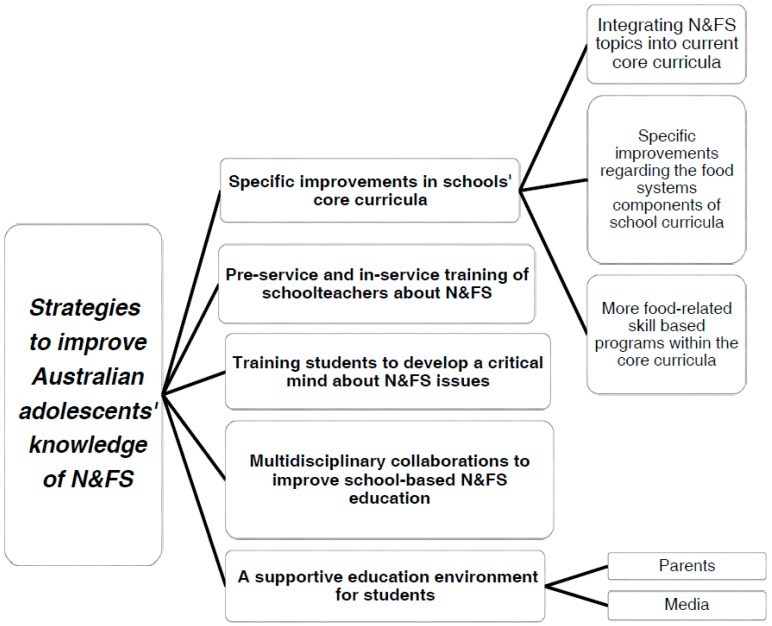
Professionals’ recommended strategies to improve Australian adolescents’ knowledge of nutrition and food systems (N&FS).

The recommended strategies to improve adolescents’ N&FS knowledge were organized into five central themes and five sub-themes described below. Identified themes are shown in Figure 1.

The interviewees from different groups of experts recommended three main strategies to improve the current core curricula of Australian schools. The first suggestion was to integrate N&FS topics into current core curricula. The second strategy was specific improvements in relation to the food systems components of school curricula. The third strategy was to increase food skills development within the core curricula. These three strategies are described separately below.

There was consensus across the different groups of interviewees that N&FS topics need to be integrated into current core curricula such as Science, Geography, History, and English Literature. Some interviewees noted that there was “very little chance” to allocate a separate subject for N&FS topics within school curricula. They stated that it was not necessary to have a separate subject for N&FS lessons because N&FS topics can be easily integrated into current core subjects. They also believed the integration of important N&FS topics within current subjects provided the opportunity for all students from across the Australia to receive an appropriate level of food-related education regardless of the schools they attend or subjects studied.

“There is very little chance of getting a new solo called nutrition subject and that is not necessary….Curriculum at schools can do this not by having a separate nutrition class, but integrating nutrition and food systems issues into the normal science curriculum…into Biological Sciences, Geography, History, English Literature and other…for example English teachers can get the students to write about food security questions…get them in geography classes to write about water and food distribution, in history classes they can look at how food supply in Australia has changed or somewhere else has been changed since world war two”.

(interviewee #21)

Most of the interviewees shared a common belief about the need for specific improvements in relation to the food systems components of school curricula, stating that the major focus of current school and community-based food education programs are to improve health outcomes. However, some experts including public health nutritionists, nutritionists, dietitians, veterinary physicians and the environmental scientist emphasized the need to educate students about food production systems and more generally about food from farm to fork, in relation to both agriculture and animal-based food products. In addition several experts also believed some animal welfare and environmental sustainability information should be incorporated into lessons addressing food production systems.

“I think that it is important to provide students with opportunities to gain an understanding and perhaps through visits to companies and projects and other things where they actually investigate how food is grown, how food is processed, how it gets to the consumer is an important part of learning about their life support systems”.

(interviewee #10)

“They need to know about what is a sustainable healthy food basket…a basket of goods needs meet some nutrition requirements and anything over that is waste. …they need to know about not using packaging…. They need to know about foods that they choose and sustainable food system and sustainable environment”.

(interviewee #7)

The home economists and most of the interviewees with nutrition backgrounds (public health nutritionists, nutritionists and dietitians) reported that food skill programs like shopping, cooking, storing foods and gardening needed to be one part of the core curricula to assist adolescents to improve their skills for everyday life. These experts focused on the importance of healthy cooking and food preparation courses for all students from primary school until school-leaving age.

“First of all it is the shopping, it’s the making a shopping list, making a healthy meal, knowing what to have in your pantry and refrigerator, seasonal ingredients so that’s the food literacy skills then the food preparation the cooking skills”.

(interviewee #6)

### 3.1. Pre-Service and In-Service Training of Schoolteachers about N&FS

The home economists and some nutritionists, public health nutritionists and dietitians frequently spoke of the critical need for training and re-training of school teachers about important N&FS topics to enable them to transfer accurate and updated information to students. Some of these interviewees noted the enrichment of school curricula with essential N&FS topics should be supported by adequate N&FS pre- and in-service training for schoolteachers.

“Teachers need to be better prepared and not assume they can teach it from no background”.

(interviewee #6)

### 3.2. Training Students to Develop a Critical Mind about N&FS Issues

Two interviewees involved in policy-making and food-related educators identified that students need to develop a broader view and a critical mind about food. These experts stated that students need to find the ability to think and discuss food-related issues in a multidimensional context. It was reported that students needed to achieve a general understating of social, political, environmental, economic and cultural aspects that are associated with food consumption, food production and food choices. Some interviewees believed students needed to learn to identify and search appropriate N&FS information sources and should learn basic research skills.

“Integrate political issues, issues of public interest, encourage and teach them to think critically and ask them to write critical argument. They need to recognize how social, economic and political determinants influence our food consumption”.

(interviewee #21)

### 3.3. Multidisciplinary Collaborations to Improve School-Based N&FS Education 

Some interviewees recognized the challenges associated with the broad and multi-disciplinary nature of N&FS topics. They reported that it was necessary to create further collaborations among schools, Departments of Education and universities (and experienced experts from different food-related disciplines) to improve N&FS education programs in schools. This was considered necessary to ensure: the identification of all essential N&FS components of school curricula; the provision of regular updates relating to the N&FS components of school curricula; the provision of appropriate education and training for schoolteachers about N&FS topics; and the planning of food-related education programs for students.

“We need to engage different educationalists to find how they can use food-related issues into current curriculum. I think we need to make sure that university level educators are having input into the school curriculum. I think, it should be cutting edge material that goes into that”.

(interviewee #8)

### 3.4. A Supportive Education Environment for Students

Interviewees from the different groups of experts stated that parents and the media need to create a more supportive environment to facilitate and support the process of educating students about N&FS. The experts’ suggestions to improve the roles of parents and media are described separately below.

*Parents:* some of the interviewees believed it was necessary to educate parents about N&FS issues in order to create a more supportive environment at home, which they considered was an important education setting for children. The purpose was to facilitate appropriate role models and to prepare well-informed food educators for the home setting. Some experts suggested changes in relation to students’ knowledge, attitude and behaviour about food-related issues are unlikely to occur without proper parental education. Building parents’ knowledge and skills was identified as a solution to overcome time constraints and overcrowded school curriculum.

“The parents, the school and the students, and teachers all are working together. That’s really important because we could be teaching students but we also need to ensure that the parents are also involved so that it’s always a team work approach. That’s important, a whole school approach so that what we’re teaching in the schools is also being underpinned and, and supported at home”.

(interviewee #15)

*Media:* some of the interviewees from different groups of experts expressed the need for more educational food-related programs in the media rather than just food related entertainment. Some experts referred to popular cooking television (TV) shows in Australia which promote cooking and eating practices that are not consistent with the Australian Dietary Guidelines’ recommendations. In addition some experts believed junk food advertising during children’s programming on Australian TV distorts children’s knowledge of appropriate eating habits. A further suggestion was for mass media to support public discourse by acknowledged food related experts.

“If there was a master chef that focused on healthy eating I think it would be a wonderful initiative. I think all the cooking shows are probably doing more damage to our food supply than anything else because there is no regard to nutrition at all. There just, you know the standard thing of butter and lots of salt, lots of dairy fat, lots of everything that I would regard as something as a special occasion so it’s kind of party food rather than everyday food. So a sort of Jamie Oliver in Australia would be a really good outcome.”.

(interviewee #4)

## 4. Discussion

This study explored experienced food professionals’ recommendations of strategies to improve Australian adolescents’ knowledge of nutrition and food systems (N&FS). Experts’ suggestions addressed five key issues including: improvements in schools’ core curricula; the importance of training school teachers about N&FS; training students to think more broadly and critically about N&FS issues; multidisciplinary collaborations to improve N&FS education programs in schools; and development of a supportive education environment for students by parents and media.

One strategy to improve the schools’ core curricula was integrating N&FS topics into current core subjects. This finding is consistent with overseas studies that identified or made reference to successful interventions that integrated nutrition lessons with other subjects such as science, language and mathematics at schools [1,6,32]. An intervention study in the United States (US) involved the integration of nutrition within other school subjects over the course of one year [6]. The findings identified the positive influence of integrative food-based curriculum on students’ nutrition knowledge in the intervention group compared to the comparison group.

The potential positive effects of an integrative curriculum on students’ knowledge was identified in the current study but to the best of our knowledge there has not been reports of any studies that have investigated the effects and practicality of a separate subject for N&FS topics. Thus it is not possible at this time for food educators and researchers to compare the relative effectiveness of an integrative N&FS-based curriculum versus a separate subject for N&FS.

Another suggested strategy to improve school curricula was more skill-based programs within the core curricula from primary school to high school. The importance of food skill programs has been supported by studies that identified the positive effects of school-based gardening, or food preparation and cooking interventions on children and adolescents’ dietary behavior [9,22,23,33]. A recent systematic review and meta-analysis of teaching approaches to improve healthy dietary behavior in primary school children [26], identified that ‘experiential learning approaches’ such as gardening, food preparation and cooking had the greatest effects compared to other approaches on increased knowledge of nutrition, reduced energy intake and increased intake and preference of fruits and vegetables [26].

The interviewees in this investigation believed there was a need for specific improvements in education about food systems in Australian schools. Unfortunately, in Australia and internationally, food systems education programs have not been considered to be necessary, essential long-term learning objectives. In contrast, food systems investigators have highlighted the importance of students’ food systems knowledge and the impacts of people’s food choices on the sustainability of the environment and the food system [15,19,34]. The connection between people’s food choices and food systems has been raised in studies focusing on the exploration of consumers’ choices in regard to: local foods [35], organic foods [16,36] animal friendly food products [37] and environmentally friendly food products [36,38]. Incorporating more food system topics within current school lessons may support adolescents’ informed food choices by not only addressing health issues but also factors such as environmental and food system sustainability, farm animal welfare, and welfare of local producers and farmers.

The current study has identified the need for training school teachers about important N&FS topics. No recent studies have reported on Australian schoolteachers’ knowledge of N&FS issues. More than one decade ago a study in Australia explored trainee home economics and physical education teachers’ knowledge, attitudes, and behaviors related to weight management, body image and eating disorders [39]. The findings revealed that while teachers had not received nutrition education, they did recommend diets to overweight teenagers despite their lack of knowledge in relation to adolescent nutritional requirements, weight management and fad diets. In addition the study reported uninformed views about eating disorders and use of inappropriate weight loss methods was identified among the studied teachers [39]. A recent study of 181 US teachers, who were responsible for the nutrition education of one million children from lower socio-economic backgrounds revealed only 3% of teachers answered four out of five nutrition knowledge questions accurately, 54% agreed that it was difficult to distinguish which nutrition information was reliable, and only 9% of teachers reported they followed healthy dietary behaviors. This study highlighted the importance of nutrition education for teachers to enable them to teach students about important nutrition issues and to improve their own dietary behaviors [40].

Overall, despite the lack of recent literature identifying schoolteachers’ knowledge of broad range of N&Fs issues, nutrition education researchers have confirmed the importance of equipping schoolteachers with adequate knowledge of nutrition to improve their willingness and confidence to teach nutrition-related lessons [41]. In addition, Worsley and colleagues in a study of food knowledge related to Australia suggested that home economists programs may influence students’ long-term food knowledge [42]. Development of specific strategies to target schoolteachers’ education needs may also be necessary. A recent cluster-randomized trial study of 20 school teachers in rural China [43] found that a comprehensive school-based nutrition program did not improve teachers’ knowledge, behavior and attitudes about nutrition. Thus teachers’ involvement in interventions that primarily target students may not be sufficient to improve their own N&FS knowledge and skills.

The interviewees involved in the current study expressed the need for media to create a supportive education environment for students. Nutrition education researchers have highlighted the important role of television in distributing nutrition education messages, for example via broadcasted food advertisements [44]. However, literature related to Australia has shown a lack of a supportive education environment of Australian television that has allocated most of the food advertisements to non-core foods (63%) [45]. The food professionals in the current investigation raised concerns about popular cooking TV shows in Australia (e.g., Masterchef), which portrayed dietary and food practices not consistent with Australian Dietary Guidelines. However, such views may be purely personal and not reflective of actual influence of such television programs. A recent Australian study identified that adult participants did not consider celebrity chefs and TV cooking shows affected their dietary intakes [46]. Young people are also high users of social media and Australian studies have shown the effects of social media on particular dietary behaviors, such as risky alcohol consumption [47]. At the same time, nutrition educators have underscored the positive potential of social media as a low-cost, quick and direct way to communicate nutrition messages [48].

In addition to media, the interviewees of current study expressed the need for parents to create a supportive education environment for their children. The importance of this issue is revealed by Australian and overseas studies, which have reported the influence of parents on their children’s dietary behavior [49,50,51,52]. For example, an Australian study found that parents influence young children’s eating habits, but often lacked the required nutrition knowledge and food skills to improve their children’s dietary behavior [49]. While this may be a useful suggestion from a school’s perspective, a more holistic approach to N&FS education, it may be unrealistic. Parents may not consider they need to be ‘educated’ about N&FS issues. Parents also may not believe they have an education role with regard to their children’s N&FS knowledge, which they may consider to be the school’s responsibility. Further research needs to address this issue.

Not all identified N&FS education strategies identified in current study have been previously reported in the literature. For example, the experts in the current study believed there is a need to develop critical thinking in students in relation to N&FS issues. However, development of critical thinking in students in relation to food consumption, food choices, food production issues or more broadly N&FS issues has not been well addressed in the existing literature. Another finding of this study was the importance of increasing multidisciplinary collaborations of schools, Departments of Education and universities. Such collaborations were identified as necessary to improve the quality and efficiency of N&FS education programs offered to students and schoolteachers.

## 5. Conclusions

The findings of this study provide important points for consideration to develop efficient and effective strategies to improve Australian adolescents’ knowledge of N&FS. The five key components included: improvements in schools’ core curricula; training schoolteachers about important N&FS topics; developing critical minds about N&FS issues; multidisciplinary actions to improve N&FS education programs in schools; and improving the positive involvements of parents and media to create a supportive education environment for students. This information will assist curriculum developers, education policy developers, and food-related educators in their endeavors to improve adolescents’ knowledge and skills of N&FS issues and, in turn, to address key public health priorities in Australia.
